# Multiscale Modeling in Computational Biomechanics: A New Era with Virtual Human Twins and Contemporary Artificial Intelligence

**DOI:** 10.3390/bioengineering12030320

**Published:** 2025-03-20

**Authors:** Tien-Tuan Dao

**Affiliations:** Univ. Lille, CNRS, Centrale Lille, UMR 9013-LaMcube-Laboratoire de Mécanique, Multiphysique, Multiéchelle, F-59000 Lille, France; tien-tuan.dao@centralelille.fr

Over the last several decades, computational biomechanics has been intensively investigated as part of the study of human body systems (musculoskeletal, cardiovascular, digestive, etc.) at multiple scales. Typically, computational biomechanics has been coupled with experimental biomechanics in order to enhance our understanding of the shape–function properties of human bodies and their causal relationships. Thus, novel biomarkers and quantitative indicators have been extracted for the support of clinical decision-making and optimization of medical devices. Recently, Virtual Human Twins (VHTs) have been introduced as a new paradigm within computational biomechanics [1]. A VHT is defined as a digital representation of a human health or disease state at different levels of human anatomy (e.g., cells, tissues, organs, or systems). The applications of VHT technology range from modeling and simulation, for the understanding of fundamental physiological and pathophysiological phenomena and processes, to the prevention, diagnosis, treatment, and follow-up of diseases in patients.

For this Special Issue, Contribution 1 (Kang et al.) developed and evaluated a VHT of the human knee, using MRI and CT data to study the changes in stress effect in the knee joint at different levels of fibular osteotomy and varus deformity. They highlighted the clinical importance of considering force distribution and the position of fibular osteotomies. Moreover, Contribution 3 (Wang et al.) developed and evaluated a VHT of a bionic knee joint, based on the geared five-bar mechanism, in order to study the influence of this new design and its associated hinge joint on the robot’s motion performance. They showed the accurate trajectory tracking capacity of the new design and a reduction in its power demand and energy consumption under a high-speed running and jumping gait.

Contribution 4 (Song et al.) developed and evaluated a VHT of the human foot for studying sports shoe complexes, showing the biomechanical relevance of the foot–shoe interaction in order to optimize footwear design. In particular, they highlighted the importance of the issue of validation when developing a VHT. With a similar interest in foot–shoe interactions, Contribution 5 (Zhu et al.) proposed a randomized crossover study to investigate the effects of running shoes with raised toe boxes. Their results may help to prevent toe injuries caused by distance running. In addition, Contribution 8 (Clemente-Suárez et al.) reviewed the field’s exploration of the neurobiological mechanisms underlying the synergistic effects of tailored nutritional strategies and exercise interventions on brain health and mental well-being. They highlighted a need for a roadmap with new bioengineered solutions in order to revolutionize both preventive and therapeutic strategies in mental health care.

Contribution 2 (Mehr et al.) developed a VHT of human scleral tissue to quantify its deformation stated under electrical stimulation. They proposed that this large differentiation could be captured by an arbitrary Lagrangian–Eulerian finite element method, integrating the transient variability of fixed charge densities with the coupling between mechanical and chemo-electrical phenomena, and their approach led to an improvement in computational accuracy.

In addition, contemporary artificial intelligence (AI) has also been investigated in many fields, ranging from computer vision to precision medicine. Different learning strategies such as deep learning, transfer learning, and reinforcement learning have been developed as the field attempts to shift from applied artificial intelligence toward general artificial intelligence, and advanced AI-driven models and tools have been developed to deal with multimodal biomedical data (scalar, text, image, signal, and video data). In this new era, the field of computational biomechanics has also been metamorphosed, integrating new AI-driven models and decision-making support mechanisms. AI technologies have been deployed to various ends, including computation speed augmentation, data interpolation/assimilation, and physics/biology augmentation (synthetic data, in silico trials, hybrid modeling). Different methodologies for the use of AI have also been proposed, spanning single-AI approaches, combination-AI approaches, and hybrid physics–AI approaches, including physics-informed, physics-augmented, and AI-embedded methods.

For this Special Issue, Contribution 6 (Nguyen et al.) developed and evaluated a new method for rapidly reconstructing a VHT of the human face from a single image using different deep learning approaches. Their obtained results showed that their proposed method may enable accurate reconstruction, both for healthy subjects and facial palsy patients. They highlighted the relevance of a representative learning database which can be virtually generated using statistical shape modeling, as suggested in the work of Contribution 7 (Tran et al.).

Computational biomechanics has faced new challenges in responding to and coupling with contemporary AI approaches. The exploration of VHTs of human structures/organs and their interactions with AI approaches open up new avenues through which we can generate new knowledge and deepen our understanding of complex physiological and pathophysiological processes. In particular, reinforcement learning has shown its capacity to explore VHTs of the face and lower limbs and design patient-specific treatment outcomes for facial rehabilitation [2] and age-related falls [3]. As a positive outcome, the field of computational biomechanics will benefit from the advancement of contemporary AI research in the future (Figure 1).

## Figures and Tables

**Figure 1:**
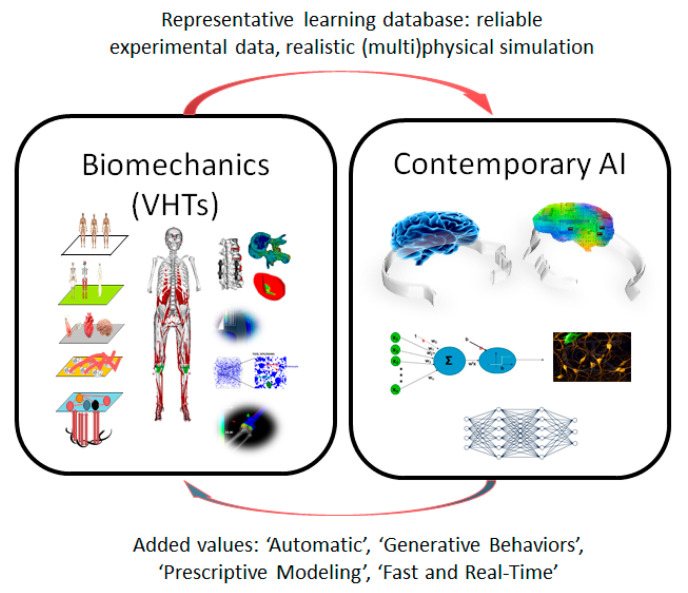
The novel AI–biomechanics coupling paradigm.

## Data Availability

Data are contained within the article.
